# Outcomes after scarf osteotomy with and without Akin osteotomy a retrospective comparative study

**DOI:** 10.1186/s13018-019-1241-7

**Published:** 2019-06-26

**Authors:** Gerhard Kaufmann, Maximilian Hofmann, Hanno Ulmer, David Putzer, Philipp Hofer, Dietmar Dammerer

**Affiliations:** 1 OFZ Innsbruck, Innrain 2 / 3.Stock, 6020 Innsbruck, Austria; 2 Orthopedic Department, Medical University of Innsbruck, Anichstrasse 35, 6020 Innsbruck, Austria; 3 Department of Medical Statistics, Informatics and Health Economics, Medical University of Innsbruck, Anichstrasse 35, 6020 Innsbruck, Austria; 4 Department of Experimental Orthopedics, Medical University of Innsbruck, Anichstrasse 35, 6020 Innsbruck, Austria

**Keywords:** Hallux valgus deformity, Radiological outcome, Scarf osteotomy, Akin osteotomy, Preoperative deformity, PDPAA, Proximal to distal phalangeal articular angle

## Abstract

**Background:**

The scarf osteotomy is a well-established surgical method for correcting a hallux valgus deformity. It is often combined with an Akin osteotomy. However, clear guidelines defining indication criteria are missing. The purpose of this study was to analyze the radiological outcome after scarf osteotomy in dependence of additional Akin osteotomy.

**Methods:**

This study included 184 patients in whom a hallux valgus deformity was corrected with a scarf osteotomy (group S), and 63 patients in whom an additional Akin osteotomy was performed (group SA). Weight-bearing radiographs were evaluated preoperatively, postoperatively, after 6 weeks, after 3 months and at a follow-up with a mean of 45.4 months. Analysis was made for the following radiological parameters: the intermetatarsal angle (IMA), the hallux valgus angle (HVA), the distal metatarsal articular angle (DMAA), the proximal to distal phalangeal articular angle (PDPAA), and the position of the sesamoids as well as the joint congruity.

**Results:**

Radiographic recurrence (HVA > 20°) was detected in 1 patient (1.6% of recurrence) in the SA group, and in 27 patients in the S group (14.7% of recurrence) at follow-up. Outcome between the two groups differed significantly showing reduced loss of HVA correction in the SA group (*p* < 0.001). The subgroup with a preoperative PDPAA above eight degrees showed significant inferiority of outcome for the S group compared to the SA group.

**Conclusion:**

Radiological outcome after scarf osteotomy is superior with concomitant Akin osteotomy. A preoperative PDPAA above eight degrees makes additional Akin osteotomy recommendable.

**Level of evidence:**

Therapeutic, Level III, retrospective comparative series

## Introduction

Scarf osteotomy is one of the most frequently used surgical methods to correct moderate to severe hallux valgus deformities [1]. In respect to the anatomical site of correction, this osteotomy is classified as a midshaft osteotomy [2], which makes the correction of severe deformities more likely. After initial description by Barouk and Weil [3, 4], several publications presenting powerful correction and good outcome with this technique have been published [5, 6]. Reduction of the complete deformity by a singular metatarsal osteotomy remains uncertain, and recurrence is a commonly occurring complication after scarf osteotomy [7]. The scarf osteotomy is a metatarsal osteotomy and does not correct a deformity of the proximal phalangeal bone, which is expressed by a pathological PDPAA (proximal to distal phalangeal articular angle). Correction of the IMA (intermetatarsal angle) can be achieved with the scarf osteotomy by shifting the first metatarsal. A varus rotation of the metatarsal head is performed to restore DMAA (distal metatarsal articular angle). Since an extended rotational maneuver could result in a bony contact of the first and the second metatarsal, correction of DMAA remains limited. In cases of a large DMAA, correction with a closing wedge osteotomy or double osteotomy might be superior. Certain radiological factors influencing outcomes have been identified so far. Most of them and their influence are discussed controversially [8, 9]. Joint congruity and soft tissue realignment have been proven as predictive factors of the outcome [10, 11].

The high rate of recurrence after scarf osteotomy [7] may be the reason why an additional Akin osteotomy is frequently performed. Nevertheless, the implementation of an additional Akin osteotomy remains a surgeon’s decision; clear guidelines for its indication are however still missing. Some authors regard the Akin osteotomy mandatory for bunion correction [12–15]. Only a few studies have presented outcome data after combined scarf Akin osteotomies so far [12, 16, 17]. The Akin osteotomy corrects a phalangeal deformity, whereas the scarf osteotomy corrects the hallux valgus deformity on the metatarsal level. After hallux valgus correction, hallux valgus interphalangeus could intraoperatively be found in many cases [18]. The authors of this study explained this finding with an underestimation of a hallux valgus interphalangeus deformity on the preoperative radiograph. Phalangeal hyperpronation might result in a change in the projection of certain angles. To assess phalangeal pathology, the hallux valgus interphalangeus angle (HIA) is frequently used, although the measurement of this angle has been shown to vary significantly [19]. In a recent publication, PDPAA has been shown to represent hallux valgus interphalangeus more precisely than HIA [20]. Figure 2 shows the projection of HIA and the PDPAA on a standing dorsoplantar radiograph. To our knowledge, no previous study has focused on the comparison between singular scarf (S) and combined scarf with Akin osteotomy (SA). To date, it remains uncertain if the high rate of recurrence is a function of the deformity or may be attributed to the surgical technique. We hypothesize that the outcome after hallux valgus correction with the scarf osteotomy may be influenced by a phalangeal pathology. In regard to this, we presume that neglection of a hallux valgus interphalangeus might result in higher recurrence after scarf osteotomy.

The purpose of our study was (1) to elucidate differences in the outcome after scarf osteotomy in respect of additional Akin osteotomy and (2) to determine radiological parameters, which require a concomitant Akin osteotomy.

## Materials and methods

Data was collected from a series of patients who underwent a hallux valgus correction by means of a scarf osteotomy at our department and from whom radiographs were available. Data was collected retrospectively. The study was approved by the local ethics committee.

Via electronic search by means of the ICD (International Statistical Classification of Diseases and Related Health Problems, WHO) and the MEL-code (benefit-related coding system of the national hospitals, National Ministry of Health) data on all patients who underwent a hallux operation in this period was collected. Using the medical chart of every listed patient, all patients with scarf osteotomy and a combination of scarf and Akin osteotomy were identified and assigned to this study. Following the definition of a hallux valgus deformity, patients with a preoperative HVA of less than 20 or an IMA of less than 10° were excluded from analysis. The aim of this exclusion was to focus on patients with adult hallux valgus deformity and to prevent falsification of the results by cases with diverging indications [21–23]. Patients with additional surgery (Weil osteotomy) on the same foot were excluded to prevent side effects on the IMA. Patients under the age of 18 years were excluded from analysis as well, since the juvenile hallux valgus deformity is regarded to be a separate pathology [24]. None of the surgeons participating in this study regarded Akin osteotomy to be necessary for every bunion correction. Despite the agreement of our institute of performing an additional Akin osteotomy in cases of hallux valgus interphalangeus, the indication to do so remained a surgeon’s decision.

**Fig. 1:**
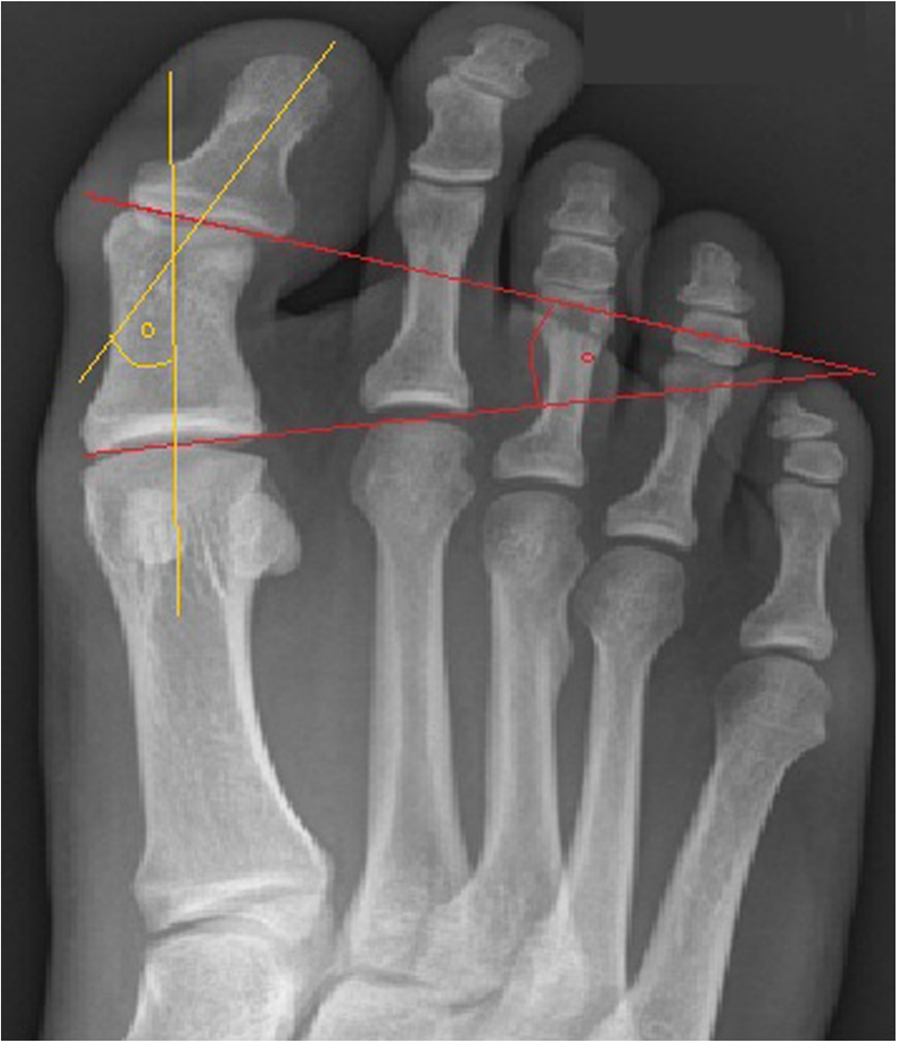
Dorsoplantar standing radiograph showing the projection of the PDPAA (red lines) and the HIA (yellow line)

In total, 184 ft of patients with a scarf (group S) and 63 with a combined Akin and scarf osteotomy (group SA) could be included to this study. The mean age at the time of surgery was 52.2 years in group S (SD 12.9, range, 21.1–81.7) and 52.0 in group SA (SD 16.2, range, 18.6–79.6) respectively. A radiographic survey was made preoperatively and postoperatively within the first 3 days, after 6 weeks, and 3 months, as it is part of our clinical routine. For all included patients we made an additional consultation of the medical chart to obtain radiographs at their latest follow-up. Radiographs were taken in anteroposterior and lateral projection with the patient in a standing position. All radiographs were read by a trained foot and ankle fellow (MH) advised by an orthopedic surgeon, who was not involved in the patients’ care. Radiographs were analyzed in a digital manner using the Icoview software (syngo.share, ITH icoserve healthcare GmbH, Siemens). The factors evaluated included (1) the hallux valgus angle (HVA), (2) the intermetatarsal angle (IMA), (3) the DMAA (by definition, a positive value for the DMAA represents a valgus tilt of the articular surface in relation to the axis of the metatarsal bone), (4) the proximal to distal phalangeal articular angle (PDPAA), which is the angle formed by tangential lines to the proximal and distal phalangeal articular surfaces (Fig. 1), (5) the position of the tibial sesamoid in relation to the midshaft axis of the first metatarsal (7-part grading system) [21], and (6) the joint congruity of the greater toe joint, which was expressed as the angle assembled by the joint lines of the metatarsal head and the proximal joint line of the proximal phalanx.

### Surgical method of scarf osteotomy

This osteotomy was performed via a 6-cm dorsomedial incision. The osteotomy was Z-shaped with the distal lever on the dorsal aspect and the proximal lever on the plantar aspect with an angle of 45°. The distal fragment was shifted laterally with a slight varus rotation to realign the joint line. Fixation was achieved with two screws (2.5 or 3.0 mm—FRS-Screw [Fusion and Reconstruction System], DePuy Orthopedics Inc., Warsaw, U.S.A), at least one of them was positioned bicortically. The prominent medial aspect of the metatarsal shaft was resected with the saw. If this piece of bone was large enough, it was grafted to the lateral side of the metatarsal and fixed with two resorbable sutures (Vicryl, Ethicon, Johnson & Johnson). The distal soft tissue procedure was performed in all cases after the dorsomedial incision of the skin and before the osteotomy was performed. Two Langenbeck hooks were placed in the intermetatarsal space 1–2, the tendon sheet of the extensor halluces was kept intact. The transverse intermetatarsal ligament was released and a T-shaped capsulotomy of the lateral joint capsula was performed to allow for reposition of the sesamoids. Closing of the medial capsula was performed with a No.1 polyglactin 910 suture (Vicryl, Ethicon, Johnson & Johnson). The skin was closed with nylon No. 3 sutures.

### Surgical method of Akin osteotomy

If an Akin osteotomy was undertaken, the dorsomedial incision was lengthened to the proximal half of the phalangeal bone. A horizontal v-shaped osteotomy was performed under the protection of the flexor and the extensor tendon of the greater toe. For Akin osteotomy, there is no guidance tool for cutting a standardized wedge. The achieved correction is a byproduct of the width of the phalangeal bone and the size of the saw blade used. Due to this, the corrective power of the Akin osteotomies in our study may have varied between the individuals. To maintain a stable hinge, violation of the lateral cortical bone was prevented. Two 1.5-mm holes were drilled with a distance of 2 mm to the osteotomy site into the proximal as well as the distal part of the phalangeal bone. Closing of the osteotomy was performed after tunneling a No.2 polyglactin 910 suture (Vicryl, Ethicon, Johnson & Johnson) through these drill holes. After tying the sutures, the skin was closed with nylon No. 3 sutures.

After surgery, patients were put in a custom-made hallux valgus shoe (Ofa GmbH, Bamberg, Germany) for 6 weeks and were allowed to exert weight on that foot. During the first 2 weeks, a sterile wound cover in the correction position of the greater toe was applied, followed by active and passive mobilization of the greater toe joint.

### Statistical method

With sample sizes of *n* = 184 (scarf) versus *n* = 63 (scarf and Akin), this study had a 92% statistical power for detecting the mean between the two groups. The difference of 0.5 standard deviations for measured parameters was achieved using independent t-tests with a 2-sided 0.05 significance level. 77% of the group fell within a standard deviation of 0.4. Sample characteristics are given as means, standard deviations, and frequencies. Comparisons of the scarf and Akin with the scarf groups with regard to the sociodemographic and clinical variables were based on Fisher’s exact tests and *t* tests for independent samples or Mann-Whitney *U* tests when data differs from normal distribution. For pairwise comparisons between groups (scarf versus scarf plus Akin) and for comparison within time points (preoperative, postoperative and follow-up) concerning the radiographic outcomes, we used *t* tests for independent samples or Mann-Whitney *U* tests and paired *t* tests or Wilcoxon tests. In addition, we used linear regression analysis to describe associations between various radiographic angles. All statistical analyses were conducted with SPSS 20.0 (International Business Machines Corporation, Armonk, NY, USA).

## Results

**Fig. 2:**
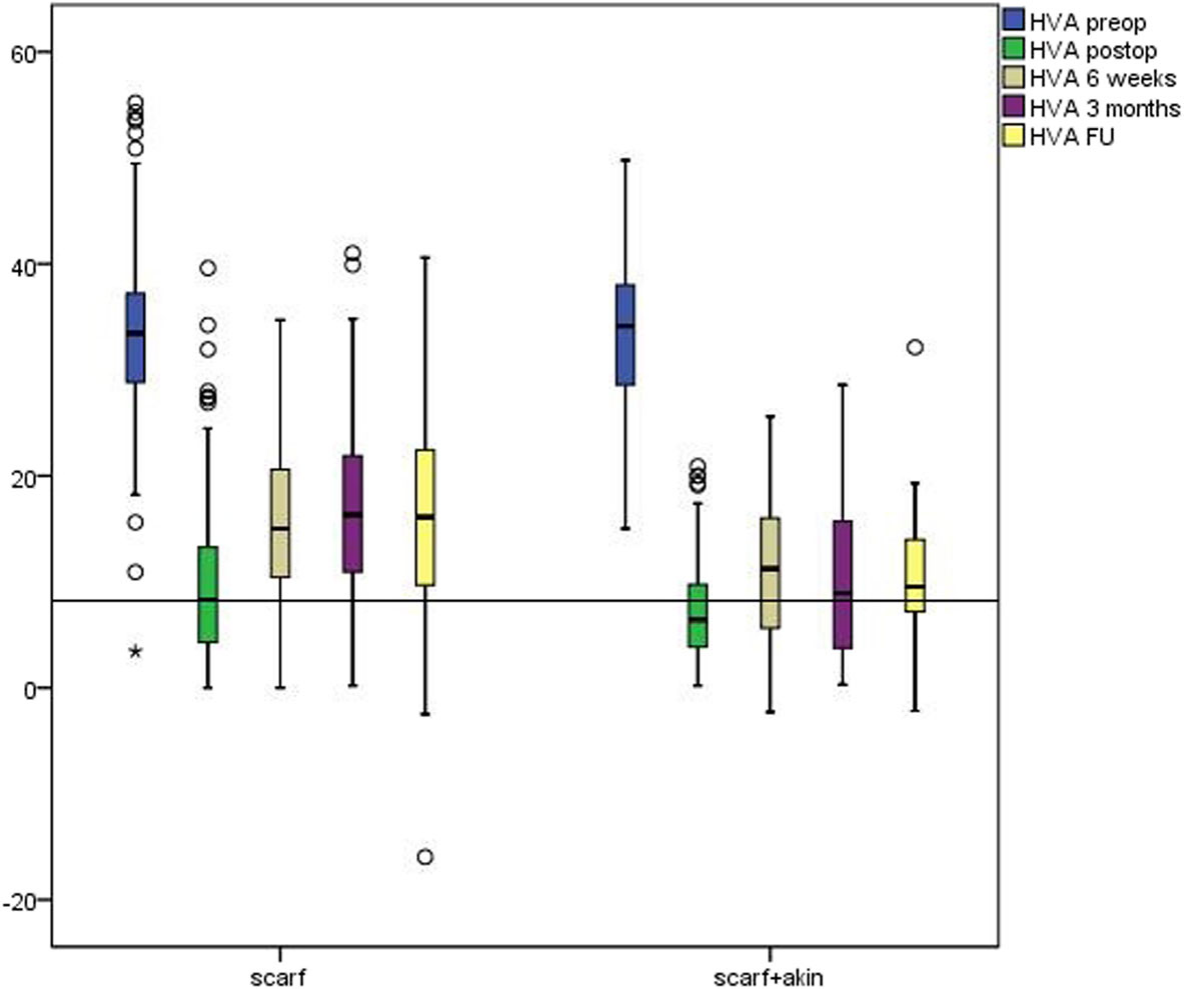
Boxplot showing HVA preoperatively, postoperatively, after 6 weeks, 12 weeks, and at follow-up for the S and the SA cohort. The black line indicates the median of the postoperative HVA of the S cohort. HVA preop, hallux valgus angle preoperative; HVA postop, hallux valgus angle postoperative; HVA 6 weeks, hallux valgus angle after 6 weeks; HVA 3 months, hallux valgus angle after 3 months; HVA FU, hallux valgus angle at follow-up

**Fig. 3:**
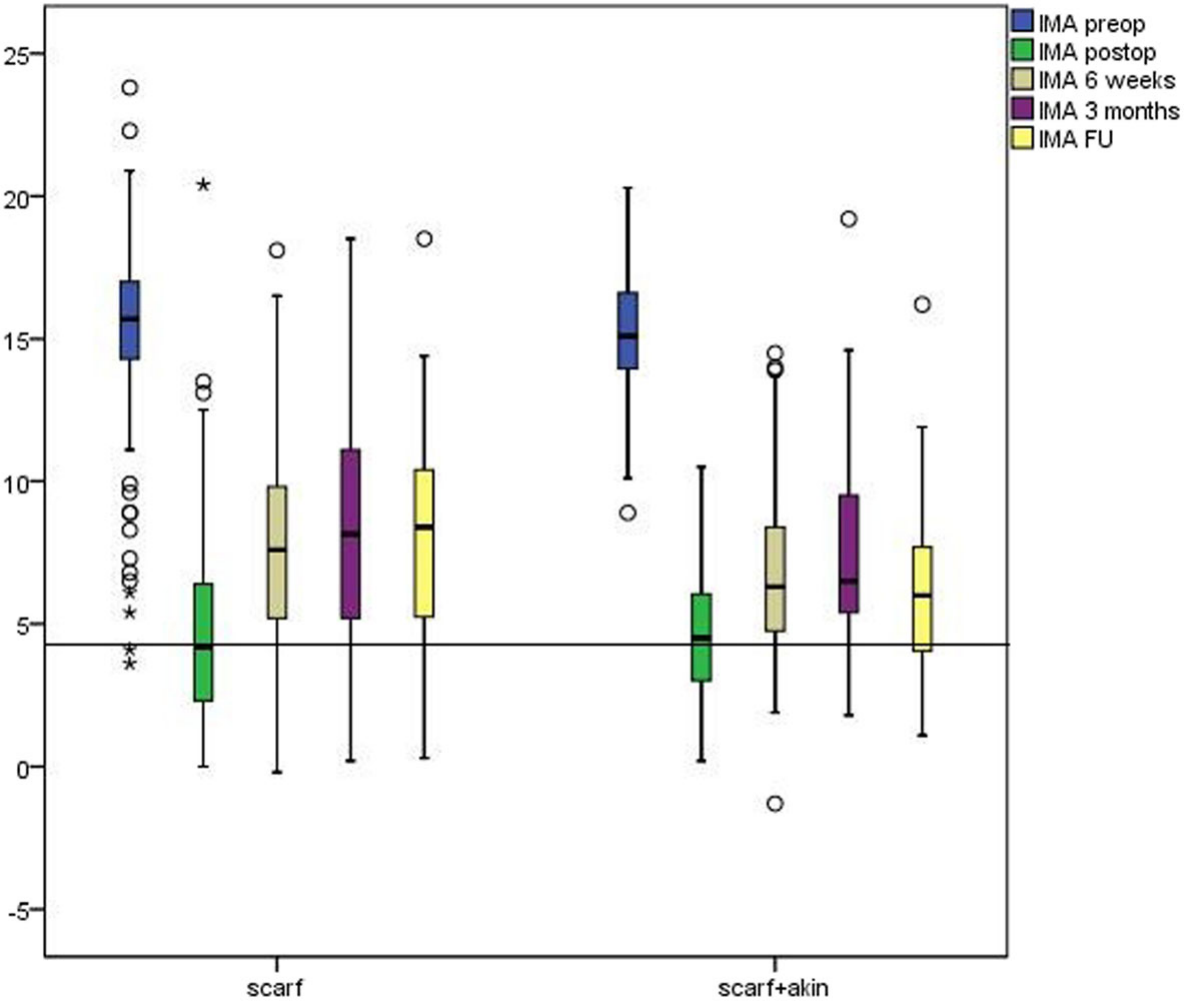
Boxplot showing IMA preoperatively, postoperatively, after 6 weeks, 12 weeks, and at follow-up for the S and the SA cohort. The black line indicates the median of the postoperative IMA of the S cohort

Between January 2002 and December 2012, we could ascertain 1117 patients with 1378 ft who underwent an isolated correction of the first ray at our department. After excluding patients with incomplete radiographic chart, additional surgery on the second ray, and other surgical methods, a total of 247 ft met the inclusion criteria (scarf osteotomy, no hallux operation in the medical history, no additional forefoot surgery on the same foot) and was applied for analysis. Sixty-three of these feet were treated with a concomitant Akin osteotomy (group SA) and 184 with a scarf osteotomy exclusively (group S). Average age at time of operation was 52.3 years ± 15.6 years with no statistical differences between the two groups. The youngest patient in the study was 18.6 years and the oldest 81.7 years of age. Twenty-three patients were male (8.9%) with a similar distribution in both groups. Table 1 summarizes the patient demographics and radiological data. Time for the follow-up examinations was 45.4 months at the mean. Preoperatively radiological data between the two groups was comparable. Only for PDPAA a difference was evident. Preoperative radiological values of both groups are presented in Table 2. Throughout follow-up improvement for PDPAA with negligible loss of correction could be found in the SA but logically not in the S group (Table 3). From pre- to postoperative, we found significant improvement in both groups for all parameters (Table 3). Loss of correction could be determined for all assessed parameters, showing differences between the two groups and favoring the SA group (Table 3). The development of HVA and IMA is pictured in Figs. 2 and 3, respectively. We found significant correction of HVA in both cohorts at all points of the survey with significantly better improvement in the SA cohort. However, we found a loss of HVA correction in both cohorts as well. The majority of correction loss could be detected from post-operative to 6 weeks with higher extent in the S group for both HVA as well as IMA. Throughout follow-up, only minor deterioration of both angles could be detected favoring the SA group (Figs. 2 and 3).

**Table 1:** Baseline (preoperative) characteristics of the S and SA osteotomy group. Preoperative characteristics and demographics of the S and SA cohort

Variable	S Group (*n* = 184)	SA Group (*n* = 63)	
Sex			.554^a^
Male	17 (8.9%)	6 (8.9%)	
Female	167 (8.9%)	57 (90.1%)	
Age (years)*	52.2 ± 15.1 (21.1–81.7)	52.0 ± 16.2 (18.6–79.6)	.774^b^
Site			.999^a^
Right	97 (52.7%)	30 (47.6%)	
Left	87 (47.3%)	33 (52.4%)	

The values are given as the number of patients, with the percentage in parentheses

*The values are given as the mean and the standard deviation, with the range in parentheses

^a^Fisher’s exact test

^b^Two-tailed, non-parametric Mann-Whitney *U* Test

*S group* scarf group, *SA group* scarf and Akin group

**Table 2:** Preoperative radiological data for scarf and combined scarf and Akin osteotomy. Preoperative radiological data of the S and SA cohort

	Scarf (S)		Scarf and Akin (SA)		
	Mean	SD	Mean	SD	*P* value
IMA preoperative	15.3	3.1	15.1	4.6	0.665
HVA preoperative	33.5	7.7	33.8	7.3	0.777
DMAA preoperative	11.8	7.0	10.3	6.5	0.119
PDPAA preoperative	6.0	3.5	10.2	5.7	0.000
Joint congruity	21.1	9.2	21.2	10.3	0.970
Sesamoids	6.1	1.0	5.9	1.2	0.343

*IMA* intermetatarsal angle, *HVA* hallux valgus angle, *DMAA* distal metatarsal articular angle*, PDPAA* proximal to distal phalangeal articular angle, *SD* standard deviation

**Table 3:** Postoperative radiological data for scarf and combined scarf and Akin osteotomy. Postoperative radiological data of the S and SA cohort

	Scarf (S)		Scarf and Akin (SA)		
	Mean	SD	Mean	SD	*P* value
IMA postoperative (degrees)	4.6	3.2	4.6	2.4	0.899
IMA 6 weeks	7.5	3.6	6.6	3.2	0.120
IMA 12 weeks	8.2	4.0	7.5	3.6	0.210
IMA FU	8.3	3.9	6.3	3.7	0.120
HVA postoperative (degrees)	9.6	7.2	7.7	5.4	0.056
HVA 6 weeks	15.5	7.1	11.0	6.4	0.000
HVA 12 weeks	16.4	7.9	10.3	7.3	0.000
HVA FU	16.6	10.5	10.4	7.6	0.019
DMAA postoperative (degrees)	7.9	5.4	6.9	4.1	0.167
0DMAA 6 weeks	6.7	6.6	6.1	6.8	0.581
DMAA 12 weeks	6.9	5.5	5.9	5.2	0.220
DMAA FU	8.7	8.3	8.1	9.5	0.804
PDPAA postoperative (degrees)	7.8	3.6	5.7	3.7	0.001
PDPAA 6 weeks	7.3	4.4	4.4	4.0	0.000
PDPAA 12 weeks	7.2	3.9	5.9	4.6	0.028
PDPAA FU	7.2	4.7	4.6	3.6	0.036
Joint congruity postoperative (degrees)	7.7	6.1	5.6	5.1	0.018
Joint congruity 6 weeks	7.6	6.3	9.5	9.2	0.087
Joint congruity 12 weeks	8.5	6.8	9.5	8.5	0.346
Joint congruity FU	8.7	7.7	8.4	7.8	0.872
sesamoids postoperative (7-part)	1.6	1.0	1.6	0.7	0.873
sesamoids 6 weeks	2.4	1.2	2.3	1.2	0.755
sesamoids 12 weeks	2.6	1.4	2.7	1.4	0.569
sesamoids FU	2.7	1.2	2.4	0.8	0.296

*IMA* intermetatarsal angle, *HVA* hallux valgus ngle*, HVA* hallux valgus angle*, DMAA* distal metatarsal articular angle*, PDPAA* proximal to distal phalangeal articular angle*, SD* standard deviation, *FU* follow up

Radiological recurrence of a hallux valgus deformity, defined a HVA surmounting 20°, could be detected in 1.6% in the SA group and 14.7% in the S group. From these patients with radiological recurrence, 7 cases in the S cohort have undergone surgical revision at our department. The patient in the SA cohort with recurrence did not undergo revision surgery so far.

**Fig. 4:**
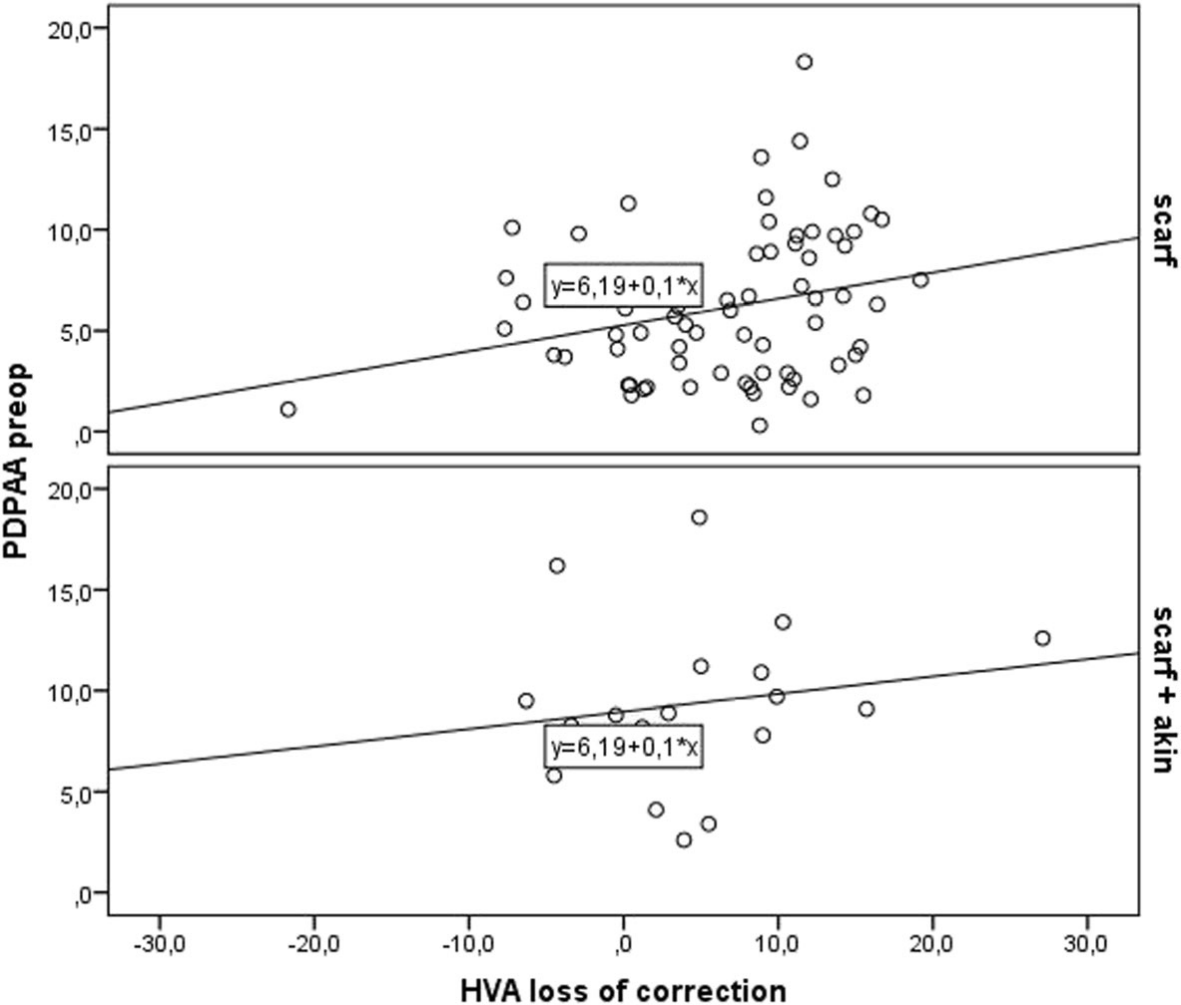
Scatterplot showing correlation of preoperative PDPAA (vertical axis) and loss of correction at follow-up (horizontal axis) for the S and the SA cohort. Black line representing regression line. Cohort S—on top, Cohort SA—below

HVA is one of the most frequently used radiological parameters to describe the hallux valgus deformity and served in our study as an outcome-determining factor. A scatterplot analysis showed a direct correlation of the amount of the preoperative PDPAA and the loss of HVA correction for both groups (S and SA). However, the correlation for the S group was stronger than for the SA group (Fig. 4). According to the detected increased loss of correction in cases of increased preoperative PDPAA, an additional survey was taken. In respect to published data, we analyzed both cohorts in regard to the preoperative PDPAA [25, 26]. Underestimation of a hallux valgus interphalangeus deformity in bunion patients is common and is expressed by an increased PDPAA after metatarsal correction. Arnold et al. recommended Akin osteotomies if PDPAA surmounts 10° [25]. However, an average deterioration of 10° of PDPAA after metatarsal osteotomy has been detected as well [26]. This increase of 2° can be ascribed to a malprojection on the preoperative radiograph. Therefore, we determined a threshold value of 8° to be more reliable. To confirm this presumption, we first assessed the S cohort with regard to a preoperative PDPAA of 8° and 10° respectively. The findings of this analysis are presented in Table 4. The calculated difference of HVA (between the subcohorts above and below the threshold value) showed significance to all points of the survey with a threshold of 8°. Calculation with 10° in contrast revealed markedly lower differences between the groups with less significance as well. The S and the SA cohort were evaluated in regard to the defined threshold value of 8°. The findings of this evaluation are summarized in Table 5 for all four subgroups. Loss of correction of HVA was detectable from postoperative to follow-up in all four subsets with significantly higher levels in the S cohort with preoperative PDPAA above eight degrees. The group below 8° experienced a loss of HVA correction of 5° in the S cohort and of 0.7° in the SA cohort. This resulted in a correction of HVA of 19.6° versus 24.6° at follow-up. In the subgroups, above 8° of preoperative PDPAA loss of correction amounted to 12.2 in the S cohort and to 3.4 degrees in the SA cohort. In consequence, HVA correction at follow-up amounted to 10.7° versus 23.2° in these cohorts. These findings demonstrate the superiority of outcome after scarf osteotomy, if a preoperative PDPAA above 8° has been corrected by an additional Akin osteotomy.

**Table 4:** Calculated difference (∆) of HVA (mean) in the scarf cohort in dependence of PDPAA between the subcohort above and below the cutoff values

	PDPAA cutoff 10°	PDPAA cutoff 8°
∆ HVA postop	4.1 (*p* = 0.072)	4.2 (p < 0.001)
∆ HVA 6 weeks	2.5 (*p* = 0.009)	4.0 (p < 0.001)
∆ HVA 12 weeks	2.3 (*p* = 0.007)	4.4 (p < 0.001)
∆ HVA FU	10.3 (*p* = 0.069)	11.4 (p < 0.001)

*HVA* hallux valgus angle, *∆ HVA* calculated difference of HVA, *PDPAA* proximal to distal phalangeal articular angle, *FU* follow up, *S* scarf

**Table 5:** Influence of preoperative PDPAA on HVA values

	PDPAA < 8°		PDPAA > 8°		
	Mean	SD	Mean	SD	*P* value
HVA preoperative (S)	32.8	7.8	35.3	7.2	0.054
HVA postoperative (S)	8.2	6.1	12.4	8.1	< 0.001
HVA 6 weeks (S)	14.1	6.0	18.1	8.5	0.001
HVA 12 weeks (S)	15.1	7.2	19.5	9.0	0.001
HVA FU (S)	13.2	8.9	24.6	9.7	< 0.001
HVA preoperative (SA)	31.8	8.2	35.0	6.6	0.096
HVA postoperative (SA)	6.5	4.6	8.4	5.7	0.190
HVA 6 weeks (SA)	10.2	6.3	11.3	6.4	0.534
HVA 12 weeks (SA)	9.5	6.9	10.7	7.5	0.530
HVA FU (SA)	7.2	5.1	11.8	8.5	0.276

*HVA* hallux valgus angle, *PDPAA* proximal to distal phalangeal articular angle*, SD* standard deviation*, FU* follow up*, SA* scarf and concomitant Akin*, S* scarf

The combination of scarf and Akin osteotomy revealed significantly better correction of the hallux valgus deformity with lower rates of loss of correction in comparison to a singular scarf osteotomy in our study. The postoperative radiographs of the S cohort and the SA cohort showed significant correction of the HVA in all points of the survey. Loss of correction was significantly reduced in the SA cohort.

## Discussion

In moderate to severe hallux valgus deformities, phalangeal pathology is a frequent finding. This deformity is meant to influence the progression of the HVA and the IMA by amplifying tractional forces on the greater toe joint. The Akin osteotomy corrects the phalangeal bone and therefore does not affect the IMA directly. In our cohort mean correction with the scarf osteotomy in our study was 15.2° for HVA and 7.4° for IMA versus 23.9° and 8.8° in the SA cohort, respectively. The achieved correction in our cohorts was higher but still comparable to that of previous studies [4, 27]. The better results of the SA cohort can be ascribed to the realigning of the phalangeal bone in combination with a good correction of the metatarsal pathology by combining a scarf with an Akin osteotomy. Other parameters like DMAA, joint congruity, and positioning of the sesamoids showed significant improvement from pre- to postoperative without detectable differences between the S and SA cohorts and without significant changes throughout follow-up. Midshaft osteotomies are associated with a higher risk of complications, such as non-union and overcorrection [28, 29]. In recent studies, high rates of recurrence have been found with the scarf technique [7, 30]. Several studies presenting outcome after scarf osteotomies or after combined Akin and scarf osteotomies have been published so far [12, 13, 31, 32]. Nevertheless, the use of an additional Akin procedure remains a surgeon’s decision to date without clear guidelines for its application. Multiple studies have presented analyses of radiological parameters with possible impact on outcome after hallux valgus correction. However, their correlation with outcome is still discussed controversially [9, 33]. One study identified joint congruity as an outcome predicting factor at least for short-term results [10]. Another publication outlined soft tissue realignment as a predictive factor as well [11].

The finding that the preoperative PDPAA affects the radiological outcome after scarf osteotomies can be regarded as the most important one of our study. We found a significantly higher loss of HVA correction surmounting 8° of preoperative PDPAA in the S cohort. In the SA cohort, in contrast, loss of correction was minor irrespective to the preoperative PDPAA. Recurrence of a hallux valgus deformity could be detected only in cases with increased preoperative PDPAA, the majority in the S cohort with 14.7% versus 1.6% in the SA cohort. Clear guidelines for recommendation of additional Akin osteotomy in hallux surgery are missing to date. Some authors recommend its application in cases of insufficient correction after the metatarsal osteotomy [18, 34]. In our department, an Akin osteotomy is performed in cases of a hallux valgus interphalangeus deformity. However, the measurement of HIA, the most commonly used angle to describe this deformity, has shown significant variation [19]. In consequence, the indication for Akin procedure is mainly a surgeon’s decision. A distinct decision tree for the application of the Akin procedure cannot be outlined for our study in regard to the retrospective study design.

In literature, correction of PDPAA above 10° has been recommended [25]. The frequent underestimation of a hallux valgus interphalangeus deformity in patients with a hallux valgus deformity has been shown intraoperatively as well [18]. An increase of PDPAA of 1.5° in patients who had undergone hallux valgus surgery without additional Akin osteotomy has been described recently [26]. The detected differences for PDPAA are meant to arise from a hyperpronation of the phalangeal bone in hallux valgus deformity resulting in a malprojection on the standing radiograph. An off axis ap view might be beneficial for detecting the real deformity of the phalangeal bone in the future. In accordance with these studies, we determined a preoperative PDPAA of 8° as the threshold value for our analysis [18, 25, 26]. We could prove 8° to be a reliable threshold compared to the previously described 10° of PDPAA.

It seems remarkable that the application of an additional Akin osteotomy has an impact on hallux valgus recurrence. Moreover, the amount of the HVA correction exceeds the achieved correction of the PDPAA. Besides the bony correction of the phalangeal pathology with the Akin osteotomy, we ascribe this finding to a change in the soft tissue balancing. The changes on tissue tension lead to an additional correcting effect and results in better HVA values. A preoperative PDPAA of more than 8° leads to significantly higher loss of correction, if an additional Akin is not performed. We ascribe this to a persisting pathological soft tissue tension in these cases. In cases below 8°, this tension seems to be negligible. Therefore, we think that the contribution of a phalangeal pathology should be taken into account in hallux valgus correction to achieve good results.

In summary, our findings point out that correction of hallux valgus interphalangeus in terms of PDPAA surmounting 8° is recommendable to prevent loss of correction after hallux valgus deformity correction. The variable outcome after scarf osteotomies in literature might be a consequence of heterogeneous study cohorts, since in most studies the pathology of the phalangeal bone has not been assessed.

### Limitations

A limitation of this study stems from the monocentric character and the retrospective nature. Another limitation of our study is that it was no a priori analysis but that it was a radiographic analysis without functional outcome assessment. Furthermore, in regard to the retrospective nature, the cohort sizes were different, lacking an appropriate control group.

The most positive aspect remains the size of our data pool.

## Conclusion

Radiological outcome in terms of reduced loss of correction after scarf osteotomy is superior with concomitant Akin osteotomy. For preoperative PDPAA of 8°, additional Akin osteotomy is highly recommended.

## Data Availability

All data generated or analyzed during this study are included in this published article. This study or contents of this study have not been published or submitted for publication elsewhere.

## Abbreviations

DMAA: Distal metatarsal articular angle
HVA: Hallux valgus angle
IMA: Intermetatarsal angle
PDPAA: Proximal to distal phalangeal articular angle
S cohort: Scarf cohort
SA cohort: Scarf and Akin cohort
SD: Standard deviation
